# Histological and Immunohistochemical Evidence in Hypothermia-Related Death: An Experimental Study

**DOI:** 10.3390/ijms26157578

**Published:** 2025-08-05

**Authors:** Emina Dervišević, Nina Čamdžić, Edina Lazović, Adis Salihbegović, Francesco Sessa, Hajrudin Spahović, Stefano D’Errico

**Affiliations:** 1 Department of Forensic Medicine, Faculty of Medicine, University of Sarajevo, 71000 Sarajevo, Bosnia and Herzegovina; emina.dervisevic@mf.unsa.ba (E.D.); adis.salihbegovic@mf.unsa.ba (A.S.); 2 Department of Pathology, Faculty of Medicine, University of Sarajevo, 71000 Sarajevo, Bosnia and Herzegovina; nina.camdzic@mf.unsa.ba (N.Č.); edina.lazovic@mf.unsa.ba (E.L.); 3 Department of Medical, Surgical and Advanced Technologies “G.F. Ingrassia”, University of Catania, 95121 Catania, Italy; 4 Urology Clinic, Clinical Center University of Sarajevo, 71000 Sarajevo, Bosnia and Herzegovina; hajrudin.spahovic@mf.unsa.ba; 5 Department of Medical, Surgical and Health Sciences, University of Trieste, 34100 Trieste, Italy; sderrico@units.it

**Keywords:** hypothermia, primary hypothermia, secondary hypothermia, alcohol, benzodiazepines, forensic pathology, immunohistochemistry

## Abstract

Hypothermia-related deaths present significant diagnostic challenges due to non-specific and often inconsistent autopsy findings. This study investigated the histological and immunohistochemical alterations associated with primary and secondary hypothermia in an experimental Rattus norvegicus model, focusing on the effects of benzodiazepine and alcohol ingestion. Twenty-one male rats were divided into three groups: control (K), benzodiazepine-treated (B), and alcohol-treated (A). After two weeks of substance administration, hypothermia was induced and multiple organ samples were analyzed. Histologically, renal tissue showed hydropic and vacuolar degeneration, congestion, and acute tubular injury across all groups, with no significant differences in E-cadherin expression. Lung samples revealed congestion, emphysema, and hemorrhage, with more pronounced vascular congestion in the alcohol and benzodiazepine groups. Cardiac tissue exhibited vacuolar degeneration and protein denaturation, particularly in substance-exposed animals. The spleen showed preserved architecture but increased erythrocyte infiltration and significantly elevated myeloperoxidase (MPO)-positive granulocytes in the intoxicated groups. Liver samples demonstrated congestion, focal necrosis, and subcapsular hemorrhage, especially in the alcohol group. Immunohistochemical analysis revealed statistically significant differences in MPO expression in both lung and spleen tissues, with the highest levels observed in the benzodiazepine group. Similarly, CK7 and CK20 expression in the gastroesophageal junction was significantly elevated in both alcohol- and benzodiazepine-treated animals compared to the controls. In contrast, E-cadherin expression in the kidney did not differ significantly among the groups. These findings suggest that specific histological and immunohistochemical patterns, particularly involving pulmonary, cardiac, hepatic, and splenic tissues, may help differentiate primary hypothermia from substance-related secondary hypothermia. The study underscores the value of integrating toxicological, histological, and molecular analyses to enhance the forensic assessment of hypothermia-related fatalities. Future research should aim to validate these markers in human autopsy series and explore additional molecular indicators to refine diagnostic accuracy in forensic pathology.

## 1. Introduction

Hypothermia is defined as a drop in core body temperature to 35 °C or below, representing a significant deviation from the normal 37 °C. It is typically categorized into three levels of severity: mild, moderate, and severe [1].

Primary hypothermia arises from direct exposure to cold conditions, where the body’s mechanisms for generating and conserving heat fail to offset core heat loss. In contrast, secondary hypothermia is associated with pre-existing medical conditions or treatments that impair the body’s ability to regulate its core temperature, such as hypothalamic disorders or drugs that disrupt thermoregulation. Prolonged exposure to cold can cause either localized (focal) or systemic (generalized) hypothermic damage and may lead to death in severe cases [2,3,4,5,6]. Fatal hypothermia is most likely to occur with extended exposure to freezing temperatures (0 °C), although it can also occur at higher temperatures under certain conditions [2].

Alcohol and benzodiazepines pose particular challenges in hypothermia-related fatalities, as both substances impair thermoregulation and increase susceptibility to cold [6]. Alcohol, for instance, induces peripheral vasodilation, accelerating heat loss, while benzodiazepines depress the central nervous system, further inhibiting the body’s adaptive responses to low temperatures [7]. These effects complicate forensic assessments, as the combined impact of hypothermia and substance use can obscure the physiological indicators typically associated with hypothermia-related deaths. Additionally, post-mortem findings may overlap with those of alcohol and drug toxicity, complicating the identification of hypothermia as the primary cause of death in such cases [8,9].

Both the very young and the elderly are particularly vulnerable to fatal outcomes from cold exposure. Infants, for example, have a larger body surface area relative to their weight, less body mass, and immature thermoregulatory systems, making them more prone to heat loss than adults. In forensic pathology, identifying hypothermia as the definitive cause of death has long been challenging, as autopsy findings can be vague, inconsistent, or even absent. Key macroscopic signs with higher diagnostic relevance were first documented in the late 19th and early 20th centuries, including frost erythema, Wischnewski spots (hemorrhagic gastric lesions), and Krjukoff’s sign. Despite numerous studies aimed at refining diagnostic criteria for hypothermic death, significant progress in this area has remained limited [10,11,12].

Forensic studies and other sources have documented various cadaveric signs potentially associated with hypothermic death, including red livor mortis, purple discoloration of the extremities, darkened blood, Wischnewski erosions in the stomach, pancreatic hemorrhage, lipid depletion in the adrenal cortex, and vacuolization of liver cells, along with other subtle indicators of hypoxia. However, there are no definitive autopsy signs of fatal hypothermia, and the morphological changes observed are generally non-specific, making it difficult to conclusively diagnose hypothermia as the cause of death [13].

The literature describes a range of atypical but frequently observed soft tissue injuries associated with fatal general hypothermia [14]. These include bite marks on the fingers, superficial scratches, minor abrasions on the backs of the hands and fingers, broken fingernails resulting from terminal self-digging behavior, and linear or striped excoriations on the face [15]. Additional findings may include facial wounds or bruises, particularly around the head, elbows, and knees, often interpreted as signs of terminal restlessness or paradoxical undressing—behaviors commonly observed in the final stages of hypothermia. Diagnosing hypothermia-related death requires a multifactorial and integrative approach. Essential components include investigation of the scene and environmental conditions, analysis of the case history, external post-mortem examination, forensic autopsy with histopathological assessment, toxicological testing, and additional laboratory analyses when necessary [16]. Only through a comprehensive evaluation of all available evidence can hypothermia be reliably determined as the cause of death and alternative or contributing factors be ruled out.

Furthermore, the behavioral and physiological responses during the terminal phase of hypothermia remain complex and incompletely understood. Continued research is warranted, particularly into the neurochemical and neurophysiological processes occurring in the brain under conditions of critical hypothermia, to enhance our understanding of the mechanisms underlying hypothermia-related deaths [17].

Histological findings linked to hypothermic fatalities have been noted in the forensic pathology literature. These include reductions in liver glycogen levels, focal necrosis in pancreatic tissue and surrounding fat, pancreatitis, vacuolation of adenocytes, fatty degeneration in the renal tubular epithelium (Armanni–Ebstein phenomenon), vacuolization in cells of the anterior pituitary, liver and kidneys, and hypoxic changes in the heart, including fatty degeneration of cardiac myocytes and hepatocytes [18,19,20,21,22]. Some researchers have found a significant correlation between Wischnewski spots and basal vacuolization of renal tubular cells, suggesting that the Armanni–Ebstein phenomenon may be the most characteristic histological marker of fatal hypothermia [11,13,18,19,23,24,25,26,27,28,29,30,31,32].

Recent research has explored the role of immunohistochemical markers in enhancing diagnostic specificity for hypothermia-related fatalities. Immunohistochemistry can help identify molecular and cellular alterations induced by hypothermia [33,34]. Incorporating these tools into forensic investigations could improve diagnostic accuracy in cases where conventional morphological findings are inconclusive.

This study aims to investigate the pathohistological changes associated with hypothermia in a controlled animal model, with a specific focus on evaluating the influence of acute alcohol and benzodiazepine ingestion on hypothermia-related fatalities. A secondary aim is to assess the potential utility of immunohistochemical markers in distinguishing primary from secondary hypothermia, with a focus on hypoxia-related changes, oxidative stress, and inflammation. By combining histological and immunohistochemical analyses, this study seeks to provide insights into the molecular mechanisms underlying hypothermia fatalities and to improve diagnostic approaches in forensic investigations.

## 2. Results

The results are presented in two sections: histological findings and immunohistochemical findings. Comparative analysis among the control (K), alcohol-treated (A), and benzodiazepine-treated (B) groups is provided, with statistical evaluation of immunopositivity scores.

### 2.1. Histological Findings

The main findings for each organ across the different groups (“K”, “A”, and “B”) are summarized in Table 1.

**Figure 1:**
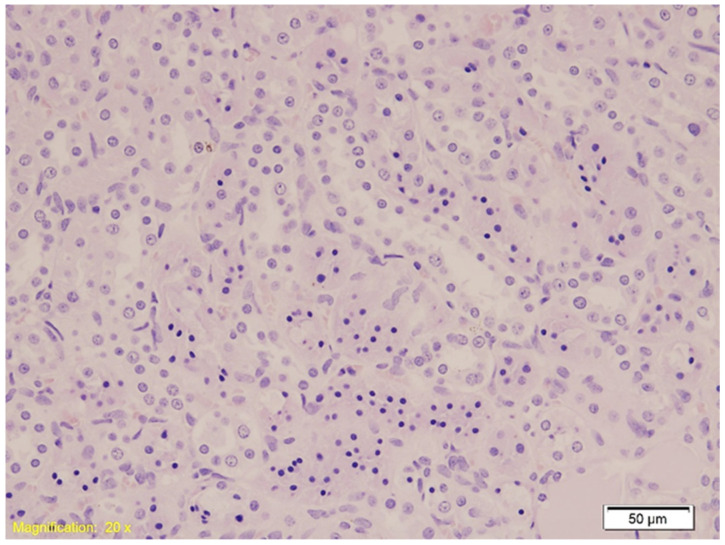
Histological section of kidney tissue showing acute tubular injury characterized by hydropic and vacuolar degeneration, congestion, and occasional lymphocytic infiltration (HE, ×20). Swollen tubular epithelial cells with clear cytoplasmic vacuoles and disrupted architecture are evident.

Histological examination of kidney tissue in the control group (K) revealed hydropic and vacuolar degeneration, vascular congestion, and occasional acute tubular injury, accompanied by minimal lymphocytic infiltration. Comparable features were observed in both the alcohol (A) and benzodiazepine (B) groups, with no significant morphological differences among them. Figure 1 demonstrates acute tubular injury with hydropic degeneration (HE, ×200).

**Figure 2:**
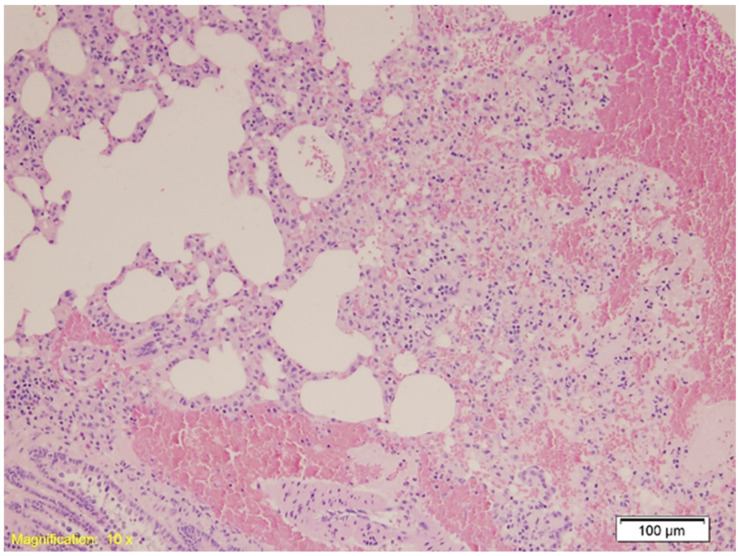
Histological section of lung tissue from Group A showing intraalveolar hemorrhage and emphysema. Alveolar spaces are filled with erythrocytes, indicative of hemorrhage, and exhibit dilation consistent with emphysematous changes (HE, ×10).

**Figure 3:**
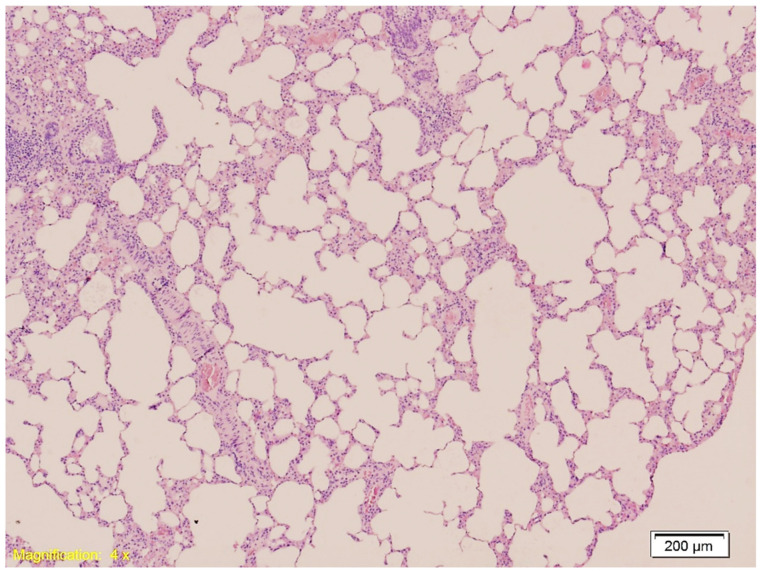
Histological section of lung tissue from Group B showing intraalveolar and intraseptal hemorrhage. Alveolar spaces are filled with erythrocytes, and the septa exhibit marked vascular congestion (HE, ×4).

In the lung, in Group K, histological examination revealed congestion, emphysema, and both intraalveolar and intraseptal hemorrhages. These changes were slightly more pronounced in Group A, although emphysema appeared reduced. Group B exhibited marked vascular congestion and extensive hemorrhage. Figure 2 shows intraalveolar hemorrhage in Group A (HE, ×10), and Figure 3 illustrates emphysema in Group B (HE, ×40).

**Figure 4:**
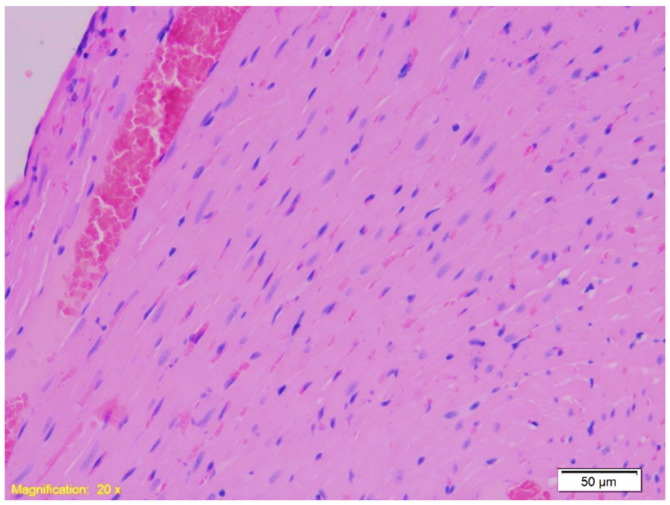
Histological section of cardiac tissue from Group A showing interstitial congestion and erythrocyte extravasation. Numerous erythrocytes are visible outside the blood vessels within the interstitial space, indicating vascular leakage and congestion (HE, ×20).

Cardiac tissues revealed interstitial congestion and erythrocyte extravasation in all groups (Figure 4). In Groups A and B, some cardiomyocytes showed vacuolar degeneration and protein denaturation.

**Figure 5:**
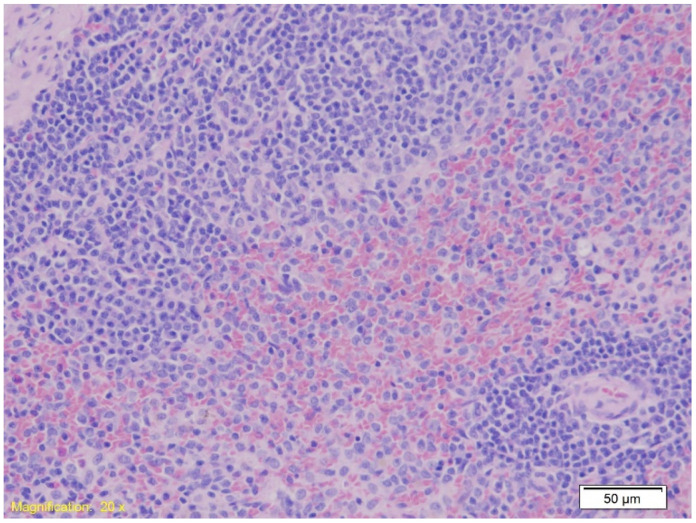
Histological section of spleen tissue showing preserved parenchymal architecture with marked erythrocyte infiltration in the red pulp (HE, ×20).

In the spleen tissue, the architecture remained preserved across all groups. Figure 5 demonstrates red pulp erythrocyte infiltration (HE, ×100).

**Figure 6:**
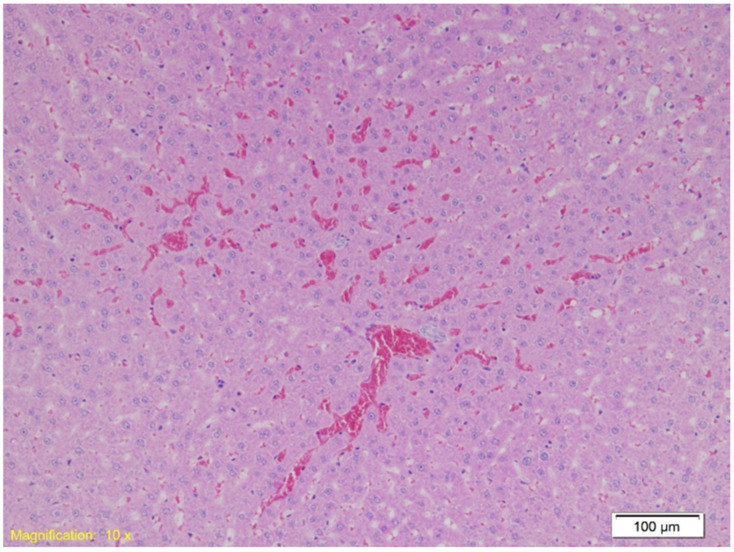
Histological section of liver tissue showing marked vascular congestion. Dilated sinusoids are filled with erythrocytes, indicating sinusoidal erythrocyte accumulation (HE, ×10).

Upon analyzing liver tissue, congestion and sinusoidal erythrocyte accumulation were present in all groups, with focal hepatocyte necrosis. Group A exhibited additional subcapsular hemorrhage. Figure 6 shows congestion and free erythrocytes in sinusoids (HE, ×10).

**Figure 7:**
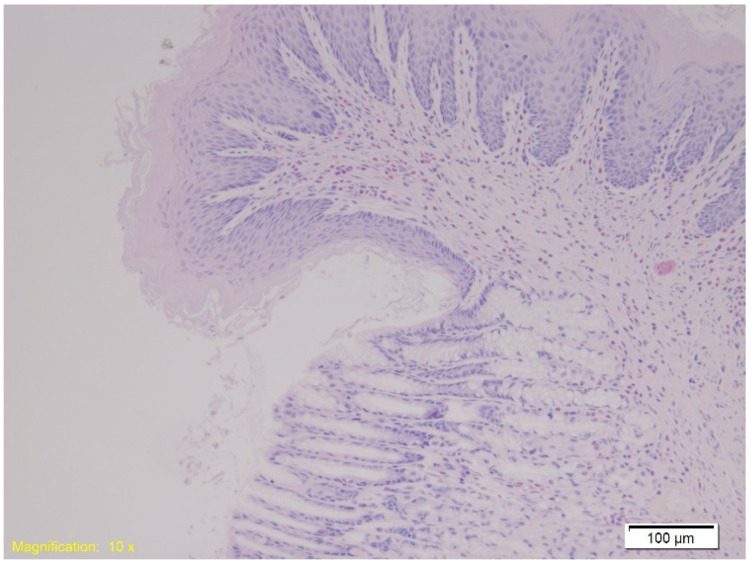
Histological section of the gastroesophageal junction shows marked subepithelial eosinophilia. Numerous eosinophils are visible within the subepithelial layer, highlighted by their characteristic red staining (HE, ×10).

In the gastroesophageal junction, muscle hypertrophy, congestion, and subepithelial eosinophilia were noted across all groups. Figure 7 depicts subepithelial eosinophilia (HE, ×200).

### 2.2. Immunohistochemical Findings

**Figure 8:**
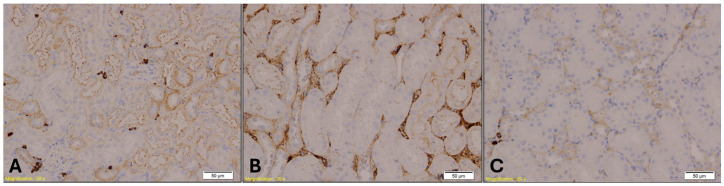
Immunohistochemical staining of kidney tissue showing E-cadherin expression in Group A (**A**), Group B (**B**), and Group K (**C**). Distal renal tubules exhibit positive membranous staining, while proximal tubules show reduced or absent expression. Magnification: ×20; scale bar: 50 µm.

E-cadherin expression in kidney tissue was assessed to evaluate epithelial integrity. Immunohistochemical analysis revealed positive membranous staining predominantly in distal renal tubules, whereas proximal tubules consistently exhibited reduced or absent expression across all experimental groups (Figure 8). Statistical analysis using the Kruskal–Wallis test showed no significant differences in E-cadherin immunopositivity among the groups (H = 0.982, *p* = 0.6121).

**Figure 9:**
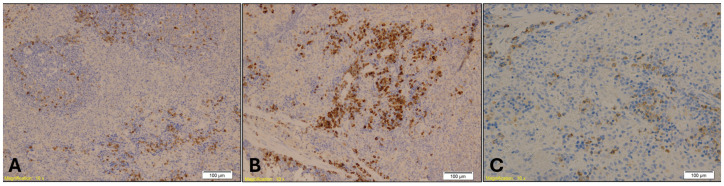
Immunohistochemical staining of spleen tissue showing MPO-positive granulocytes in Group A (**A**), Group B (**B**), and Group K (**C**). Numerous MPO-positive cells, indicated by brown staining, are visible within the red pulp in Group B, while Group A shows sparse staining and Group K displays a moderate number of positive cells. Magnification: ×10; scale bar: 100 µm.

MPO staining in spleen tissue revealed increased granulocyte infiltration in Groups A and B compared to Group K (Figure 9). The Kruskal–Wallis test confirmed a statistically significant difference in MPO expression among the groups (H = 11.22, *p* = 0.004), with mean rank scores of 8.21 for Group A, 17.00 for Group B, and 7.79 for Group K. Dunn’s multiple comparison test with Bonferroni correction indicated that MPO immunopositivity in Group B was significantly higher than in both Group A (*p* = 0.002) and Group K (*p* = 0.001), while no significant difference was observed between Group A and Group K (*p* = 0.89).

**Figure 10:**
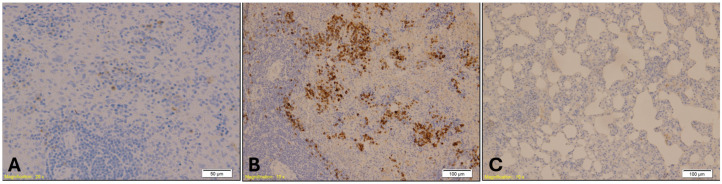
Immunohistochemical staining of lung tissue showing MPO-positive granulocytes. Group B (Benzodiazepine, **B**) displays elevated neutrophilic infiltration, indicated by dense brown staining within alveolar and interstitial spaces (magnification: ×10; scale bar: 100 µm). Group A (**A**) shows sparse MPO-positive cells (magnification: ×20; scale bar: 50 µm), while Group K (**C**) exhibits a reduced number of MPO-positive granulocytes (magnification: ×10; scale bar: 100 µm).

MPO staining in lung tissue revealed elevated neutrophilic infiltration in the benzodiazepine group, with reduced granulocyte density observed in the control group(Figure 10). The Kruskal–Wallis test indicated a highly significant difference in MPO expression among the groups (H = 16.29, *p* = 0.00029), with mean rank scores of 5.5 for Group A, 17.71 for Group B, and 9.79 for Group K. Dunn’s multiple comparison test with Bonferroni correction showed that MPO immunopositivity in Group B was significantly higher than in both Group A (*p* = 0.00003) and Group K (*p* = 0.004), while no significant difference was found between Group A and Group K (*p* = 0.08).

**Figure 11:**
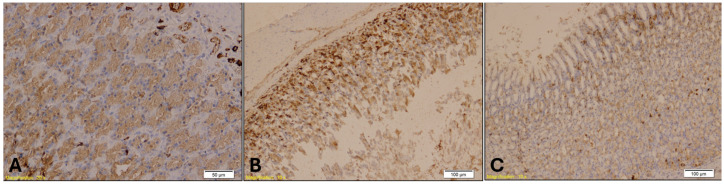
CK7 immunohistochemical staining of the gastroesophageal junction showed that CK7 immunopositivity in Groups A (**A**, magnification: ×20; scale bar: 50 µm) and B (**B**, magnification: ×10; scale bar: 100 µm) was significantly higher than in Group K (**C**, magnification: ×10; scale bar: 100 µm), while no significant difference was observed between Groups A and B.

CK7 immunostaining of the gastroesophageal junction revealed epithelial stress, particularly in the treated groups. Loosely packed mucous-secreting glands were observed in Group B (Figure 11). Statistical analysis showed a significant difference in CK7 expression in the gastroesophageal junction among the groups (H = 8.83, *p* = 0.0121). Dunn’s multiple comparison test with Bonferroni correction revealed that CK7 immunopositivity in Groups A and B was significantly higher than in Group K (A vs. K, *p* = 0.02; B vs. K, *p* = 0.005). No significant difference was observed between Group A and Group B (*p* = 0.64).

**Figure 12:**
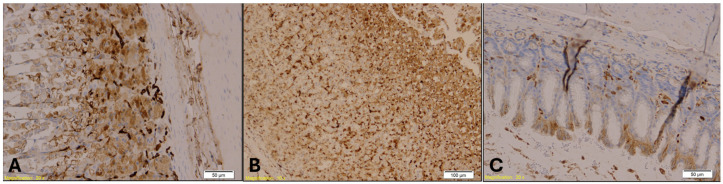
CK20 immunohistochemical staining of the gastroesophageal junction. Group A (Panel **A**; magnification: ×20; scale bar: 50 µm) and Group B (Panel **B**; magnification: ×10; scale bar: 100 µm) show strong CK20 immunopositivity in the glandular epithelium, indicated by brown staining. Group K (Panel **C**; magnification: ×10; scale bar: 50 µm) displays markedly reduced CK20 expression.

CK20 immunostaining of the gastroesophageal junction also revealed epithelial disruption, with strong immunopositivity observed in Group B (Figure 12). A similar trend of CK7 was noted for CK20 expression across the groups. The Kruskal–Wallis test indicated a highly significant difference in CK20 expression among the groups (H = 16.46, *p* = 0.00027). Post hoc analysis using Dunn’s multiple comparison test with Bonferroni correction showed that CK20 immunopositivity was significantly higher in both Group A (*p* = 0.001) and Group B (*p* = 0.0001) compared to Group K. However, no significant difference was found between Groups A and B (*p* = 0.47).

## 3. Discussion

This study was designed to investigate the morphological changes associated with primary and secondary hypothermia, specifically in the context of alcohol and benzodiazepine exposure, using an albino Rattus norvegicus model. The findings offer valuable insights into the pathology of hypothermia and its interaction with these substances, providing potential forensic markers that may help distinguish between different types of hypothermia-related fatalities. This information is particularly relevant for forensic pathologists tasked with determining the cause of death in cases where hypothermia and substance use are suspected.

Diagnosing hypothermia as the cause of death remains challenging in forensic pathology due to the lack of specific and consistent macro- and microscopic findings. While the presence of frost erythema, Wischnewski spots, bloody discoloration of knee joint fluid, synovial membrane bleeding, and basal vacuolization of renal tubular cells are considered significant indicators of fatal hypothermia, their absence does not necessarily exclude the diagnosis. Post-mortem examinations—both macroscopic and microscopic—provide valuable information about the body’s response to cold stress and the metabolic changes following cold exposure. However, alcohol intoxication interferes with the body’s cold adaptation mechanisms, complicating the diagnosis of hypothermia. Although emerging techniques such as immunohistochemistry, post-mortem imaging, and molecular pathology show promise, they currently do not offer conclusive, pathognomonic signs of fatal hypothermia.

At the molecular level, hypothermia induces oxidative stress by disrupting mitochondrial function, leading to the overproduction of reactive oxygen species (ROS) [35]. These ROS can damage cellular components—including lipids, proteins, and DNA—contributing to tissue injury observed in hypothermic conditions [36]. Furthermore, hypothermia activates apoptotic pathways, particularly through the intrinsic (mitochondrial) pathway [37]. This involves the upregulation of pro-apoptotic proteins such as Bax and the activation of caspases, including caspase-3, which mediate programmed cell death [38]. Inflammatory signaling is also modulated during hypothermia. The nuclear factor kappa B (NF-κB) pathway, a key regulator of inflammation, may be suppressed, leading to altered cytokine profiles such as reduced TNF-α and IL-6 levels [39]. This immunosuppressive effect may explain the decreased MPO-positive granulocytes observed in lung and spleen tissues. Hypoxia-inducible factors (HIFs), particularly HIF-1α, are stabilized under cold-induced hypoxic conditions. HIF-1α regulates genes involved in angiogenesis, metabolism, and cell survival, and its expression may contribute to the adaptive responses seen in hypothermic tissues [40].

In the kidneys, all groups exhibited varying degrees of hydropic and vacuolar degeneration, congestion, and occasional acute tubular injury, characterized by increased cytoplasmic eosinophilia, apical cell swelling, and pyknotic nuclei. These morphological features align with findings from studies on hypothermia-induced kidney injury, where cellular swelling and cytoplasmic eosinophilia are common due to intracellular fluid shifts and hypoxic damage [29,30]. However, focal tubular dilation and eosinophilic casts observed in the tubules are more commonly associated with hypoxic kidney injuries than with hypothermic fatalities alone, suggesting additional stress from prolonged cold exposure in this model. These features are significant in forensic contexts, as they support the hypothesis of secondary hypothermia influenced by external factors. In cases where hypothermia overlaps with alcohol or drug toxicity, these tubular signs may serve as useful indicators for pathologists, especially in deaths suspected to involve compromised renal function [31,32].

Epithelial cadherin (E-cadherin) is a transmembrane protein whose extracellular domain is involved in intercellular adhesion and polarity maintenance. It is expressed in nearly all epithelial cells, with membranous expressions considered normal. Abnormal expressions are characterized by loss or reduction in membranous staining [41].

Unlike rabbit and mouse kidneys, where E-cadherin is expressed in all tubular segments, in monkey, dog, rat, and human kidneys, E-cadherin is moderately to strongly expressed in distal tubules but absent in proximal tubules [42,43,44]. In our study, all groups, both control and experimental, showed a loss of E-cadherin expression in proximal tubules and moderate positivity in distal tubules. No differences in expression were observed among the groups, as similar morphological findings were present in all kidney specimens. Statistical analysis showed no significant difference in E-cadherin expression among the groups, indicating that renal epithelial integrity was similarly affected across conditions.

In all groups, lung tissue demonstrated congestion, emphysema, focal atelectasis, and intraalveolar and intraseptal hemorrhage. However, congestion and hemorrhagic signs were slightly more pronounced in the alcohol (A) and benzodiazepine (B) groups. These findings are consistent with previous studies reporting that hypothermia compounded by alcohol use tends to exacerbate lung congestion due to vasodilation and increased susceptibility to microvascular damage [31,32]. Similarly, benzodiazepines, which depress respiratory and circulatory functions, can increase the risk of hypoxia and hemorrhagic injury, contributing to the pronounced lung congestion observed in these groups [45,46]. Emphysematous changes were most prominent in the control (K) group, suggesting a relatively pure hypothermic response. Statistical analysis confirmed a highly significant difference in MPO expression among the groups. Dunn’s multiple comparison test with Bonferroni correction revealed that MPO immunopositivity in lung tissue was significantly higher in Group B compared to both Group A and Group K, whereas no significant difference was found between Group A and Group K. These findings suggest that MPO expression in lung tissue may serve as a sensitive immunohistochemical marker for identifying hypothermia-related deaths associated with benzodiazepine intoxication, reflecting an enhanced neutrophilic response and oxidative stress. This marker could be particularly useful in forensic settings where traditional histological signs are inconclusive or when substance use is suspected but not yet confirmed toxicologically.

Hypothermia modulates the host immune response, particularly affecting the number and activity of neutrophils, leading to their downregulation [47]. Myeloperoxidase (MPO) is a myeloid marker that strongly stains neutrophils and variably stains other granulocytes. It is a non-specific marker of neutrophil activity and inflammation [48]. Based on this, a decreased number of MPO-positive granulocytes is expected in the lungs of rats that died from hypothermia. In our study, we observed a reduced number of MPO-positive granulocytes in lung specimens from the control (K) group and the experimental groups (A and B).

Although ethanol induces vasodilation, significant effects on lung tissue typically require chronic abuse. In individuals with long-term alcohol use, it contributes to lung dysfunction by compromising the airway epithelial barrier, impairing the binding and phagocytic functions of alveolar macrophages, and disrupting mucociliary clearance in the respiratory tract. Collectively, these effects reduce pathogen clearance and weaken the immune response, facilitating infection [49].

In all groups, the heart exhibited congestion and erythrocyte extravasation into the interstitium. In the alcohol group, additional signs of cardiomyocyte cytoplasmic granulation and vacuolar degeneration were observed, likely due to protein denaturation. Similar findings have been reported in previous studies, where alcohol was shown to impair mitochondrial function in cardiac cells under hypothermic stress, resulting in protein denaturation [50,51,52]. Benzodiazepine-related cases showed occasional vacuolar degeneration [53,54], which may be attributed to both hypothermic stress and the cardiovascular depressant effects of benzodiazepines. These findings are forensically relevant, as protein denaturation and vacuolar degeneration in cardiac tissue may suggest secondary hypothermia induced by alcohol or benzodiazepines. When combined with other systemic signs, these markers may help forensic pathologists differentiate pure hypothermia from cases complicated by substance use.

In the spleen, all groups showed preserved parenchymal architecture with pronounced erythrocyte infiltration in the red pulp, a sign of hypoxia likely exacerbated by hypothermia. Although spleen findings are less specific to substance influence, they are consistent with systemic hypoxic changes frequently reported in the hypothermia literature. Additionally, hypothermia has been shown to induce platelet activation in human spleen [55].

The increased granulocyte counts observed in the spleens of intoxicated rats that died from hypothermia, compared to non-intoxicated rats under the same conditions, may be explained by several mechanisms. Diazepam has been shown to induce histological changes in the spleen, liver, and kidneys of experimental rats, prompting adaptive responses aimed at mitigating toxic effects. This adaptation may result in an enhanced immune response [56]. Granulocyte levels in systemic circulation are influenced by multiple factors, including their removal by macrophages in the bone marrow stroma and the marginal zones of the liver and spleen under normal conditions, as well as their migration from the bloodstream to tissue sites during inflammation. Alcohol may affect each of these processes differently [57]. In our study, the Kruskal–Wallis test confirmed a statistically significant difference in MPO expression among the groups. Particularly, Dunn’s multiple comparison test with Bonferroni correction revealed that MPO immunopositivity in Group B was significantly higher than in both Group A and Group K, while no significant difference was observed between Group A and Group K. These findings suggest that MPO expression in the spleen may serve as a valuable immunohistochemical marker for identifying hypothermia-related deaths associated with benzodiazepine intoxication, reflecting a heightened inflammatory response potentially linked to drug-induced immune modulation.

Intoxication often triggers a robust immune response characterized by granulocyte recruitment, primarily neutrophils, as part of the innate defense mechanism. The spleen, as a central organ for immune cell storage and activation, exhibits increased granulocyte accumulation in response to systemic inflammation caused by toxins [58]. Furthermore, toxins can act as stressors that stimulate granulopoiesis through cytokine and chemokine production, particularly granulocyte-colony stimulating factor (G-CSF), increasing circulating granulocyte levels, many of which localize to the spleen [59]. Intoxication can also impair microcirculation, promoting granulocyte retention in vascular beds, including splenic sinusoids. Hypothermia exacerbates this effect by further slowing circulation and enhancing cell sequestration. Additionally, toxic substances can directly damage splenic tissue, releasing damage-associated molecular patterns (DAMPs), which recruit granulocytes to the site of injury as part of the inflammatory repair response [60]. The combination of systemic intoxication and hypothermia amplifies the inflammatory response through overlapping stress pathways. Hypothermia itself induces mild inflammation, which, when combined with the pro-inflammatory effects of intoxication, significantly enhances granulocyte recruitment to the spleen.

The elevated granulocyte count in the red pulp of the spleens from intoxicated animals may result from a combination of localized tissue damage, inflammatory responses, and systemic effects of toxic substances. This accumulation likely represents a defensive mechanism aimed at limiting damage and eliminating harmful agents. This may also explain the reduced number of MPO-positive granulocytes observed in the red pulp of the spleens in the control (K) group.

Liver findings revealed extensive congestion across all groups, with free erythrocytes present in the sinusoidal spaces and focal necrosis, particularly pronounced in the alcohol group. Similar findings of liver congestion and necrosis under hypothermic stress have been reported in previous studies. Alcohol-induced hypothermia may accelerate hepatic necrosis due to its vasodilatory effects and the associated increase in metabolic stress [61]. The presence of subcapsular hemorrhage in the alcohol group further supports this mechanism, as these regions are especially vulnerable to rapid temperature fluctuations and vasodilation. In forensic investigations, pronounced congestion, focal necrosis, and subcapsular hemorrhage in the liver may serve as important indicators of alcohol involvement in hypothermia-related deaths.

Prolonged cold exposure can lead to systemic hypoxia due to peripheral vasoconstriction and reduced blood flow to non-essential organs, including the gastrointestinal tract. Hypoxia triggers cellular injury, inflammation, and tissue responses such as congestion and hypertrophy. Studies suggest that hypothermia induces stress-related morphological changes in tissues, as observed in gastroesophageal specimens [12]. In our study, all groups exhibited muscle layer hypertrophy, congestion, and marked subepithelial eosinophilia in the gastroesophageal junction, consistent with hypoxic stress induced by prolonged cold exposure. Although not pathognomonic, these changes may help distinguish hypothermia cases from other causes of death involving hypoxic injury. Because hypothermia-related deaths often lack external signs of trauma or systemic disease, histopathological findings become crucial. The combination of gastroesophageal congestion, muscle hypertrophy, and eosinophilia may help differentiate hypothermia-induced hypoxia from other causes, such as asphyxia or ischemia secondary to cardiac arrest [11,52]. In forensic cases, particularly when other gastrointestinal injuries are absent, findings of gastroesophageal congestion and eosinophilia may provide additional evidence supporting hypothermia as the cause of death.

Immunohistochemical analysis revealed the expression patterns of CK7 and CK20, epithelial markers commonly used in forensic pathology to aid tissue characterization. While CK7 and CK20 are primarily employed to identify the origin of epithelial cells in neoplastic lesions, their detection in this context may help confirm epithelial integrity and support a non-neoplastic, hypoxia-related etiology [62]. Although not pathognomonic, these markers may assist in distinguishing hypothermia from other causes of death involving hypoxic injury. Statistical analysis showed a significant difference in CK7 expression in the gastroesophageal junction among the groups. Dunn’s multiple comparison test with Bonferroni correction revealed that CK7 immunopositivity in Groups A and B was significantly higher than in Group K, while no significant difference was observed between Group A and Group B. A comparable trend was observed for CK20 expression. These findings suggest that CK7 and CK20 may serve as useful indicators of epithelial stress in hypothermia-related deaths, particularly when influenced by alcohol or benzodiazepine intoxication. In forensic cases where hypothermia may have been influenced by substance use, toxicological analysis remains essential to determine the presence, concentration, and potential interactive effects of alcohol and benzodiazepines. Comprehensive toxicological investigations should include an analysis of blood, urine, liver, and stomach contents to accurately quantify substance levels and establish a timeline of ingestion. Alcohol can suppress shivering, reduce core heat production, and impair judgment, while benzodiazepines exacerbate central nervous system depression and may induce respiratory inhibition [63]. Understanding these pharmacodynamic effects enables forensic pathologists to differentiate between primary hypothermia and secondary hypothermia influenced by pharmacological agents.

Additionally, genetic testing for pharmacogenomic and pharmacogenetic markers is increasingly important in evaluating individual dose–response variability. Variants in genes such as CYP2C19 and CYP3A4, which are involved in benzodiazepine metabolism, can lead to altered drug clearance, with slow metabolizers experiencing prolonged drug activity and more pronounced sedative effects. Similarly, genetic differences in alcohol dehydrogenase (ADH) and aldehyde dehydrogenase (ALDH) enzymes may affect alcohol metabolism rates, with some individuals experiencing amplified effects due to delayed clearance. In forensic contexts, pharmacogenetic information can help explain why certain individuals exhibit heightened sensitivity to drugs or alcohol, particularly under hypothermic conditions [64,65].

This study has several limitations. First, the semi-quantitative scoring method used for immunohistochemical evaluation, while widely adopted, may introduce a degree of subjectivity. Although scoring was performed by experienced observers, inter-observer variability cannot be entirely excluded. Second, the sample size may limit the statistical power and generalizability of the findings. Third, as this is an animal study, caution is warranted when extrapolating the results to human cases. Humans may exhibit different metabolic and physiological responses to hypothermia, particularly in the context of alcohol and benzodiazepine metabolism, which involves complex pharmacokinetic interactions. Finally, the absence of pathognomonic histological signs of hypothermia remains a diagnostic challenge, as noted in the previous literature. Therefore, the findings should be interpreted within the broader context of case-specific evidence and in conjunction with other forensic indicators.

## 4. Materials and Methods

### 4.1. Experimental Design

This investigation was designed as a randomized, prospective, and experimental study using an albino Rattus norvegicus model to examine the effects of benzodiazepines and alcohol on hypothermia. A total of 21 male rats (3.5 months old, weighing 200–240 g) were obtained from the Veterinary Faculty, University of Sarajevo (VFUNSA). The animals were randomly assigned to one of three groups using a computer-generated randomization process:

- Control Group (K) (*n* = 7): Exposed to hypothermic conditions without any drug treatment, serving as a model for primary hypothermia.
- Benzodiazepine Group (B) (*n* = 7): Administered diazepam (5 mg/kg body weight) daily in the morning for two weeks prior to hypothermic exposure, modeling secondary hypothermia induced by benzodiazepine use.
- Alcohol Group (A) (*n* = 7): Administered red wine (Vranac, 14.0% alcohol, 10 mL/kg body weight) once daily in the morning for two weeks before hypothermic exposure, simulating secondary hypothermia caused by alcohol intake.

As a pilot study, the focus was on detecting large effect sizes, in accordance with the principles of the 3Rs for ethical research. The sample size for each group was calculated to detect a significant effect size (Cohen’s d = 2) with an α level of 0.05 and 80% power. An additional 15% was added to account for potential dropouts.

### 4.2. Animal Welfare and Ethical Approval

The study was conducted at VFUNSA and approved by the institutional ethics committee (registration number: 07-03-161-2/23). All procedures adhered to the ethical standards for laboratory animal care established by the Federation of European Laboratory Animal Science Associations (FELASA) and followed the ARRIVE guidelines [33,65].

All animals were from the same litter and were housed under the supervision of the Veterinary Faculty of the University of Sarajevo, in compliance with animal care and preservation guidelines. Prior to the experiment, the rats were acclimatized for 7 days in a controlled environment with standard ventilated cages and sawdust bedding. Environmental conditions were maintained at a 12 h light/dark cycle, a temperature of 20–22 °C, and 40–60% humidity. During this period, animals were provided with commercial laboratory feed (Pellet, Mixed Nutrition for Laboratory Animals) and had ad libitum access to water. Animal welfare was closely monitored throughout the study.

### 4.3. Hypothermia Induction and Monitoring

The control group (K) received no treatment and was maintained under normal conditions for two weeks before hypothermia induction. In the experimental groups (A and B), hypothermia was induced after two weeks of alcohol or diazepam administration, during which the animals were considered intoxicated.

Each rat was anesthetized with intramuscular ketamine (10%, 100 mg/mL, 1.2 mL/kg, Ketaminol^®^, MSD Animal Health, Unterschleissheim, Germany). A temperature probe was inserted 5 cm into the esophagus to continuously monitor core body temperature using a Physitemp Thermalert TH-8 thermometer (Physitemp Instruments, Clifton, NJ, USA), with an accuracy of ±0.1 °C.

The rats were placed on wooden boards with their heads elevated above the water and immersed in pre-cooled water at 7 °C. Water temperature was continuously monitored using a submerged probe to ensure consistency. The procedure continued until the animals succumbed to hypothermia, and survival times were recorded.

### 4.4. Histological Investigation

Following euthanasia, multiple organ samples (kidney, lung, heart, liver, spleen, and gastroesophageal junction) were collected and immediately fixed in 10% neutral-buffered formalin. After fixation, tissues were processed through a graded ethanol series (70%, 80%, 95%, and 100%) and cleared in xylene. Paraffin-embedded sections (4 µm thick) were stained with hematoxylin and eosin (H&E) for routine histological evaluation.

Histological analysis was performed using a light microscope (BX40, Microscope Central, Olympus, Tokyo, Japan) by two independent pathologists (N.Č., E.L.), blinded to group assignments. Morphological changes were documented and photographed. Discrepancies in interpretation were resolved by consensus. The evaluation focused on identifying features such as degeneration, necrosis, congestion, hemorrhage, and inflammatory infiltrates across the different experimental groups.

### 4.5. Immunohistochemical Investigation

Immunohistochemical (IHC) staining was performed using the indirect biotin–streptavidin–horseradish peroxidase (HRP) method to evaluate molecular and cellular responses to hypothermia and substance exposure. The following primary monoclonal antibodies were used, with each selected for its relevance to hypoxia, inflammation, or epithelial integrity:

- E-cadherin (mouse monoclonal, 1:200)—Kidney: E-cadherin is a transmembrane protein involved in epithelial cell adhesion and polarity. Its expression is typically preserved in distal renal tubules but reduced in proximal segments. Loss or reduction in E-cadherin staining may reflect epithelial stress or ischemic injury, making it a useful marker for assessing hypothermia-induced renal damage.
- Myeloperoxidase (MPO) (rabbit monoclonal, 1:100)—Lung and Spleen: MPO is a non-specific marker of neutrophilic infiltration and oxidative stress. It is used to evaluate inflammatory responses and immune activation, which may be altered in hypothermia and further modulated by alcohol or benzodiazepine exposure.
- Cytokeratin 7 (CK7) (rabbit monoclonal, 1:100)—Gastroesophageal Junction: CK7 is an intermediate filament protein expressed in epithelial cells, particularly in glandular and transitional zones. Its expression helps assess epithelial integrity and stress responses in hypothermia-related gastrointestinal injury.
- Cytokeratin 20 (CK20) (mouse monoclonal, 1:150)—Gastroesophageal Junction: CK20 complements CK7 in characterizing epithelial differentiation and stress. Its expression pattern can help distinguish hypothermia-induced epithelial changes from other causes of mucosal injury.

Species-specific secondary antibodies (goat anti-rabbit or goat anti-mouse IgG conjugated to HRP; Vector Laboratories) were applied at a dilution of 1:200. Visualization was achieved using 3,3′-diaminobenzidine (DAB) as the chromogen. Negative controls were prepared by omitting the primary antibody, and positive controls included rat spleen (MPO), intestinal epithelium (E-cadherin), liver (CK7), and intestine (CK20).

Two independent pathologists (N.Č., F.S.), blinded to group allocation, evaluated the immunostained slides. Immunoreactivity was assessed semi-quantitatively based on the intensity and extent of brown DAB staining. The following scoring system was used:

o (−): No staining.
o (+): Weak staining.
o (++): Moderate staining.
o (+++): Strong staining.
o (++++): Very strong staining.

Mean immunopositivity scores were calculated for each group and organ. This approach enabled comparative analysis of the expression patterns across experimental conditions, particularly in relation to hypothermia-induced tissue stress, inflammation, and epithelial disruption.

### 4.6. Statistical Analysis

Immunohistochemical staining results were evaluated semi-quantitatively by two independent pathologists, blinded to group assignments. Immunopositivity was scored based on the intensity and extent of brown DAB staining using a five-tier ordinal scale: (−) = 0, (+) = 1, (++) = 2, (+++) = 3, and (++++) = 4. For each antibody and organ, scores were recorded for all animals in the three experimental groups: control (K), alcohol-treated (A), and benzodiazepine-treated (B).

Statistical comparisons of immunopositivity scores among the three groups were performed using the non-parametric Kruskal–Wallis test followed by Dunn’s multiple comparison test with Bonferroni correction. This test is appropriate for ordinal data and small sample sizes. A *p*-value < 0.05 was considered statistically significant. All analyses were conducted using Python v. 3.12.4 (SciPy library), and the results were summarized by antibody and tissue type.

## 5. Conclusions

This study highlights the complex interplay between hypothermia, alcohol, and benzodiazepines, and their distinct morphological effects on organ tissues. Using a Rattus norvegicus model, we identified tissue-specific markers—such as increased pulmonary congestion, cardiac vacuolar degeneration, and focal hepatic necrosis—that may serve as indicators of secondary hypothermia influenced by alcohol or benzodiazepine exposure. Importantly, immunohistochemical analysis provided statistically significant evidence of altered inflammatory and epithelial responses. MPO expression was significantly elevated in both lung and spleen tissues of benzodiazepine-treated animals, suggesting enhanced neutrophilic activity and oxidative stress. CK7 and CK20 immunopositivity in the gastroesophageal junction was significantly higher in both alcohol- and benzodiazepine-treated groups compared to the controls, indicating epithelial stress and hypoxia-related changes. These findings support the potential utility of these markers in differentiating primary hypothermia from cases complicated by pharmacological agents.

The results underscore the importance of integrating histological, immunohistochemical, and toxicological analyses in forensic investigations of hypothermia-related deaths. Future research should aim to validate these markers in human autopsy series, assess their specificity across differential diagnoses, and explore additional molecular pathways involved in cold-induced tissue injury. Such efforts are essential to refine forensic protocols and improve the accuracy of cause of death determinations in complex, substance-related hypothermic fatalities.

## Figures and Tables

**Table 1:** The main findings are presented for each organ and each tested group.

Organ	Group K (Control)	Group A (Alcohol)	Group B (Benzodiazepine)
Kidney	Hydropic and vacuolar degeneration, congestion; occasional acute tubular injury (eosinophilia, swelling, pyknotic nuclei); minimal lymphocytic infiltrate in interstitium.	Similar findings as Group K; occasional acute tubular injury and lymphocytic infiltrate.	Similar findings as Group K; occasional acute tubular injury and lymphocytic infiltrate.
Lungs	Congestion, emphysema, focal atelectasis, and intraalveolar and intraseptal hemorrhage, with emphysema most pronounced.	Slightly increased congestion, intraalveolar and intraseptal hemorrhage; emphysema less pronounced.	Similar to Group A with pronounced congestion, intraalveolar and intraseptal hemorrhage; less emphysema.
Heart	Congestion and erythrocyte extravasation in interstitium.	Similar to Group K, with some cardiomyocytes showing finely granular cytoplasm and vacuolar degeneration, suggesting protein denaturation.	Similar to Group K with occasional vacuolar degeneration and protein denaturation in cardiomyocytes.
Spleen	Preserved architecture with strong erythrocyte infiltration in red pulp.	Similar findings as Group K.	Similar findings as Group K.
Liver	Strong congestion with free erythrocytes in sinusoidal spaces; occasional hepatocyte necrosis with cytoplasmic granulation (potential protein denaturation).	Strong congestion, free erythrocytes in sinusoids; focal hepatocyte necrosis, subcapsular hemorrhage zones.	Strong congestion with similar necrosis and cytoplasmic granulation (suggesting protein denaturation).
Gastro-esophagus	Muscle layer hypertrophy, congestion, marked subepithelial eosinophilia.	Similar findings as Group K.	Similar findings as Group K.

## Data Availability

The raw data supporting the conclusions of this article will be made available on request to the first author E.D. (mail to: emina.dervisevic@mf.unsa.ba).

## Abbreviations

The following abbreviations are used in this manuscript:

ADH	Alcohol Dehydrogenase
ALDH	Aldehyde Dehydrogenase
CK7	Cytokeratin 7
CK20	Cytokeratin 20
E-cadherin	Epithelial cadherin
G-CSF	Granulocyte-Colony Stimulating Factor
HIF-1α	Hypoxia-Inducible Factor 1 alpha
IL-6	Interleukin 6
MPO	Myeloperoxidase
NF-κB	Nuclear Factor kappa B
ROS	Reactive Oxygen Species
TNF-α	Tumor Necrosis Factor alpha

## References

[1] Soar J., Perkins G.D., Abbas G., Alfonzo A., Barelli A., Bierens J.J.L.M., Brugger H., Deakin C.D., Dunning J., Georgiou M. (2010). European Resuscitation Council Guidelines for Resuscitation 2010 Section 8. Cardiac Arrest in Special Circumstances: Electrolyte Abnormalities, Poisoning, Drowning, Accidental Hypothermia, Hyperthermia, Asthma, Anaphylaxis, Cardiac Surgery, Trauma, Pregnancy, Electrocution. Resuscitation.

[2] Nixdorf-Miller A., Hunsaker D.M., Hunsaker J.C. (2006). Hypothermia and Hyperthermia Medicolegal Investigation of Morbidity and Mortality from Exposure to Environmental Temperature Extremes. Arch. Pathol. Lab. Med..

[3] Lim C., Duflou J. (2008). Hypothermia Fatalities in a Temperate Climate: Sydney, Australia. Pathology.

[4] Teresiński G., Buszewicz G., Ma̧dro R. (2005). Biochemical Background of Ethanol-Induced Cold Susceptibility. Leg. Med..

[5] Zachariah S.B., Zachariah A., Ananda R., Stewart J.T. (2000). Hypothermia and Thermoregulatory Derangements Induced by Valproic Acid. Neurology.

[6] Kreuzer P., Landgrebe M., Wittmann M., Schecklmann M., Poeppl T.B., Hajak G., Langguth B. (2012). Hypothermia Associated with Antipsychotic Drug Use: A Clinical Case Series and Review of Current Literature. J. Clin. Pharmacol..

[7] Gordon C.J. (2010). Response of the Thermoregulatory System to Toxic Insults. Front. Biosci.-Elite.

[8] Michaud K., Romain N., Giroud C., Brandt C., Mangin P. (2001). Hypothermia and Undressing Associated with Non-Fatal Bromazepam Intoxication. Forensic. Sci. Int..

[9] Zonnenberg C., Bueno-de-Mesquita J.M., Ramlal D., Blom J.D. (2017). Hypothermia Due to Antipsychotic Medication: A Systematic Review. Front. Psychiatry.

[10] Dolinak D., Matshes E., Lew E.O. (2005). Forensic Pathology: Principles and Practice.

[11] Madea B., Tsokos M., Preuß J. (2009). Death Due to Hypothermia Morphological Findings, Their Pathogenesis and Diagnostic Value. Forensic Pathology Reviews.

[12] Turk E.E. (2010). Hypothermia. Forensic Sci. Med. Pathol..

[13] Payne-James J., Byard R.W. (2024). Hypothermia. Forensic and Legal Medicine: Clinical and Pathological Aspects.

[14] Chudakov A.Y., Tolmachev I.A., Kuznetsova A.A. (2025). Uncharacteristic Soft Tissue Injuries Detectable in Cases of Death from General Accidental Hypothermia. Forensic Med. Expert..

[15] Simmonds M., Llewellyn A., Walker R., Fulbright H., Walton M., Hodgson R., Bojke L., Stewart L., Dias S., Rush T. (2025). Anti-VEGF Drugs Compared with Laser Photocoagulation for the Treatment of Proliferative Diabetic Retinopathy: A Systematic Review and Individual Participant Data Meta-Analysis. Health Technol. Assess..

[16] Dettmeyer R.B. (2014). The Role of Histopathology in Forensic Practice: An Overview. Forensic Sci. Med. Pathol..

[17] You J.S., Kim J.Y., Yenari M.A. (2022). Therapeutic Hypothermia for Stroke: Unique Challenges at the Bedside. Front. Neurol..

[18] Mack T., Parai J.L., Milroy C.M. (2024). Establishing Vitreous Glucose and Beta-Hydroxybutyrate Thresholds to Assist in the Diagnosis of Hypothermia. Forensic Sci. Int..

[19] Hejna P., Zátopková L., Tsokos M. (2012). The Diagnostic Value of Synovial Membrane Hemorrhage and Bloody Discoloration of Synovial Fluid (“inner Knee Sign”) in Autopsy Cases of Fatal Hypothermia. Int. J. Leg. Med..

[20] Fineschi V., Neri M., Di Donato S., Pomara C., Riezzo I., Turillazzi E. (2006). An Immunohistochemical Study in a Fatality Due to Ovarian Hyperstimulation Syndrome. Int. J. Leg. Med..

[21] Pennisi G., Torrisi M., Cocimano G., Esposito M., Salerno M., Sessa F. (2022). Vitality Markers in Forensic Investigations: A Literature Review. Forensic Sci. Med. Pathol..

[22] Salerno M., Cocimano G., Roccuzzo S., Russo I., Piombino-Mascali D., Márquez-Grant N., Zammit C., Esposito M., Sessa F. (2022). New Trends in Immunohistochemical Methods to Estimate the Time since Death: A Review. Diagnostics.

[23] Preuß J., Lignitz E., Dettmeyer R., Madea B. (2007). Pancreatic Changes in Cases of Death Due to Hypothermia. Forensic Sci. Int..

[24] Preuß J., Dettmeyer R., Lignitz E., Madea B. (2004). Fatty Degeneration in Renal Tubule Epithelium in Accidental Hypothermia Victims. Forensic Sci. Int..

[25] Palmiere C., Mangin P. (2013). Postmortem Biochemical Investigations in Hypothermia Fatalities. Int. J. Leg. Med..

[26] Preuss J., Dettmeyer R., Lignitz E., Madea B. (2006). Fatty Degeneration of Myocardial Cells as a Sign of Death Due to Hypothermia versus Degenerative Deposition of Lipofuscin. Forensic Sci. Int..

[27] Ishikawa T., Miyaishi S., Tachibana T., Ishizu H., Zhu B.L., Maeda H. (2004). Fatal Hypothermia Related Vacuolation of Hormone-Producing Cells in the Anterior Pituitary. Leg. Med..

[28] Doberentz E., Madea B. (2017). Microscopic Examination of Pituitary Glands in Cases of Fatal Accidental Hypothermia. Forensic Sci. Res..

[29] Yamada S., Shimomura Y., Ohsaki M., Fujisaki A., Tsuruya K., Iida M. (2010). Hypothermia-Induced Acute Kidney Injury in a Diabetic Patient with Nephropathy and Neuropathy. Intern. Med..

[30] Yoon H.J., Kim M.C., Park J.W., Yang M.A., Lee C.B., Sun I.O., Lee K.Y. (2014). Hypothermia-Induced Acute Kidney Injury in an Elderly Patient. Korean J. Intern. Med..

[31] Vahdatpour C., Sussman A., Mahr T. (2019). A Case Report of Severe Hypothermia Complicated by Acute Respiratory Distress Syndrome. Respir. Med. Case Rep..

[32] Mehta A.J., Guidot D.M. (2017). Alcohol and the Lung. Alcohol Res..

[33] Hleșcu A.A., Grigoraș A., Ianole V., Amalinei C. (2024). Advanced Diagnostic Tools in Hypothermia-Related Fatalities—A Pathological Perspective. Diagnostics.

[34] Elmsjö A., Ward L.J., Horioka K., Watanabe S., Kugelberg F.C., Druid H., Green H. (2024). Biomarker Patterns and Mechanistic Insights into Hypothermia from a Postmortem Metabolomics Investigation. Sci. Rep..

[35] Zhao R.Z., Jiang S., Zhang L., Yu Z. (2019). Bin Mitochondrial Electron Transport Chain, ROS Generation and Uncoupling (Review). Int. J. Mol. Med..

[36] Juan C.A., de la Lastra J.M.P., Plou F.J., Pérez-Lebeña E. (2021). The Chemistry of Reactive Oxygen Species (Ros) Revisited: Outlining Their Role in Biological Macromolecules (DNA, Lipids and Proteins) and Induced Pathologies. Int. J. Mol. Sci..

[37] Song S.-J., Wu G.-C., Yi L., Liu X., Jiang M.-M., Zhang X.-C., Yin Z.-F., Gu W., Ruan Y. (2025). Heat Shock Proteins in Hypothermia: A Review. Front. Mol. Biosci..

[38] Hussar P. (2022). Apoptosis Regulators Bcl-2 and Caspase-3. Encyclopedia.

[39] Ageeva T., Rizvanov A., Mukhamedshina Y. (2024). NF-ΚB and JAK/STAT Signaling Pathways as Crucial Regulators of Neuroinflammation and Astrocyte Modulation in Spinal Cord Injury. Cells.

[40] Bakleh M.Z., Al Haj Zen A. (2025). The Distinct Role of HIF-1α and HIF-2α in Hypoxia and Angiogenesis. Cells.

[41] Canas-Marques R., Schnitt S.J. (2016). E-Cadherin Immunohistochemistry in Breast Pathology: Uses and Pitfalls. Histopathology.

[42] Nouwen E.J., Dauwe S., Van Der Biest I., De Broe M.E. (1993). Stage- and Segment-Specific Expression of Cell-Adhesion Molecules N-CAM, A-CAM, and L-CAM in the Kidney. Kidney Int..

[43] Prozialeck W.C., Edwards J.R. (2007). Cell Adhesion Molecules in Chemically-Induced Renal Injury. Pharmacol. Ther..

[44] Lee S.Y., Han S.M., Kim J.E., Chung K.Y., Han K.H. (2013). Expression of E-Cadherin in Pig Kidney. J. Vet. Sci..

[45] Kienitz R., Kay L., Beuchat I., Gelhard S., von Brauchitsch S., Mann C., Lucaciu A., Schäfer J.H., Siebenbrodt K., Zöllner J.P. (2022). Benzodiazepines in the Management of Seizures and Status Epilepticus: A Review of Routes of Delivery, Pharmacokinetics, Efficacy, and Tolerability. CNS Drugs.

[46] Wang S.H., Chen W.S., Tang S.E., Lin H.C., Peng C.K., Chu H.T., Kao C.H. (2019). Benzodiazepines Associated with Acute Respiratory Failure in Patients with Obstructive Sleep Apnea. Front. Pharmacol..

[47] Lim C.M., Kim M.S., Ahn J.J., Kim M.J., Kwon Y., Lee I., Koh Y., Kim D.S., Kim W.D. (2003). Hypothermia Protects against Endotoxin-Induced Acute Lung Injury in Rats. Intensive Care Med..

[48] Gaines P., Chi J., Berliner N. (2005). Heterogeneity of Functional Responses in Differentiated Myeloid Cell Lines Reveals EPRO Cells as a Valid Model of Murine Neutrophil Functional Activation. J. Leukoc. Biol..

[49] Traphagen N., Tian Z., Allen-Gipson D. (2015). Chronic Ethanol Exposure: Pathogenesis of Pulmonary Disease and Dysfunction. Biomolecules.

[50] Hoek J.B., Cahill A., Pastorino J.G. (2002). Alcohol and Mitochondria: A Dysfunctional Relationship. Gastroenterology.

[51] Manzo-Avalos S., Saavedra-Molina A. (2010). Cellular and Mitochondrial Effects of Alcohol Consumption. Int. J. Environ. Res. Public Health.

[52] Piano M.R. (2017). Alcohol’s Effects on the Cardiovascular System. Alcohol Res..

[53] Leducq N., Bono F., Sulpice T., Vin V., Janiak P., Le Fur G., O’Connor S.E., Herbert J.M. (2003). Role of Peripheral Benzodiazepine Receptors in Mitochondrial, Cellular, and Cardiac Damage Induced by Oxidative Stress and Ischemia-Reperfusion. J. Pharmacol. Exp. Ther..

[54] Surinkaew S., Chattipakorn S., Chattipakorn N. (2011). Roles of Mitochondrial Benzodiazepine Receptor in the Heart. Can. J. Cardiol..

[55] Horioka K., Tanaka H., Okaba K., Yamada S., Ishii N., Motomura A., Inoue H., Alkass K., Druid H., Yajima D. (2021). Hypothermia Causes Platelet Activation in the Human Spleen. Thromb. Res..

[56] Grahovac J., Ivanković M., Dekić R., Paraš S. (2022). Effects of Diazepam on Hematological and Histological Parameters in Rats/In Vivo and Unbiased Stereological Investigation. Acta Vet..

[57] Shi X., DeLucia A.L., Bao J., Zhang P. (2019). Alcohol Abuse and Disorder of Granulopoiesis. Pharmacol. Ther..

[58] Medzhitov R. (2008). Origin and Physiological Roles of Inflammation. Nature.

[59] Manz M.G., Boettcher S. (2014). Emergency Granulopoiesis. Nat. Rev. Immunol..

[60] Rock K.L., Latz E., Ontiveros F., Kono H. (2010). The Sterile Inflammatory Response. Annu. Rev. Immunol..

[61] Hyun J., Han J., Lee C., Yoon M., Jung Y. (2021). Pathophysiological Aspects of Alcohol Metabolism in the Liver. Int. J. Mol. Sci..

[62] Chu P.G., Weiss L.M. (2002). Keratin Expression in Human Tissues and Neoplasms. Histopathology.

[63] Gerostamoulos D., Schumann J. (2022). Forensic Toxicology: Overview and Applications. Encyclopedia of Forensic Sciences: Volume 1–4.

[64] Nunno N.D., Esposito M., Argo A., Salerno M., Sessa F. (2021). Pharmacogenetics and Forensic Toxicology: A New Step towards a Multidisciplinary Approach. Toxics.

[65] European Council of Legal and Forensic Medicine Harmonization of Medico-Legal Autopsy. https://eclm.eu/en/documents/harmonization-of-medico-legal-autopsy-protocol/.

